# Case Report: De Clerambault's Syndrome in Dementia With Lewy Bodies

**DOI:** 10.3389/fpsyt.2021.665868

**Published:** 2021-06-10

**Authors:** Takashi Suehiro, Yuto Satake, Mamoru Hashimoto, Manabu Ikeda

**Affiliations:** Department of Psychiatry, Osaka University Graduate School of Medicine, Suita, Japan

**Keywords:** de Clerambault's syndrome, erotomania, dementia, dementia with Lewy bodies, delusion

## Abstract

**Background:** Erotomania, also known as de Clerambault's syndrome, is characterized by the delusion that a person has fallen in love with the patient. It occasionally appears secondary to psychiatric disorders and organic brain diseases. However, there have been no reports on cases secondary to dementia with Lewy bodies (DLB).

**Case Presentation:** The patient was an 83-year-old woman who lived alone. Mild cognitive impairment appeared at the age of 82 years. Soon after, she had the delusional conviction that her family doctor was in love with her. Her symptoms, such as gradually progressive cognitive impairment, cognitive fluctuations, and parkinsonism, indicated DLB. She was treated with a small dose of antipsychotic agents.

**Conclusions:** This case report suggests the possibility of de Clerambault's syndrome during the early stages of DLB. Further investigations are required to clarify the mechanism and treatment of de Clerambault's syndrome in patients with DLB.

## Introduction

Erotomania (de Clerambault's syndrome) is a relatively rare disorder, characterized by the delusion that a person is in love with the patient (1). The object of the delusion commonly has a higher social status than the patient and usually remains unchanged (1). The epidemiology of the disorder is unclear (2). The pure form of de Clerambault's syndrome cannot be explained by any other psychiatric disorders and a pre-existing psychiatric disorder is associated with the onset of the syndrome in its secondary form (3). Schizophrenia is reportedly the most frequent psychiatric comorbidity in the secondary form (4). Moreover, there are few reports on de Clerambault's syndrome in the clinical course of dementia (5), such as Alzheimer's disease (AD) (2, 6, 7), vascular dementia (8), and frontotemporal dementia (9).

Dementia with Lewy bodies (DLB) is the second most common form of degenerative dementia after AD (10). While delusions, such as delusion of theft and misidentification, are highly prevalent in patients with dementia, they are more frequent in patients with DLB (4). In addition, delusions in such patients are relatively various. For instance, delusional jealousy is observed most frequently in patients with DLB (11). However, there are no reports on de Clerambault's syndrome in patients with DLB. Herein, we report a case of de Clerambault's syndrome that appeared in the early stages of DLB.

## Case Description

The patient was an 83-year-old woman. She had been receiving treatment for hypertension and constipation for more than 20 years. However, she had no other medical history, including psychiatric disorders. There was no family history of psychiatric disorders or neurodegenerative disorders. Following graduation from high school, she began working in a nightclub. She got married in her twenties and had a daughter. She divorced a few years later. Following her daughter gaining employment, she started living alone. She was on public income support during her visit to our clinic. She gradually felt a lack of motivation for outdoor activities at the age of 82 years. Simultaneously, she started facing difficulty with housework and complained of mild amnesia. A few months later, she informed her daughter about the delusional thought that the family doctor drew her blood to kill her. Despite the delusion of persecution, she continued visiting the clinic. Her daughter pointed out that her thought was delusional as it was impossible. Despite all evidence to the contrary, it remained unchanged. However, the delusion suddenly changed a month later, without any specific cause. She believed that her family doctor had fallen in love with her and proposed marriage to her. The delusional conviction seemingly strengthened with time. Moreover, she gradually made up her mind to accept the proposal. Considering the gradual progression of cognitive impairment and apathy, her daughter proposed living together. She refused her daughter's proposal and continued living alone because she was convinced that she would live with her family doctor in the near future. Her daughter recommended that she visit a memory clinic. Although she did not have any insight into her delusional beliefs, she was aware of her cognitive impairment. Therefore, she visited our memory clinic and was admitted to our hospital for examination and treatment at the age of 83 years.

**Figure 1:**
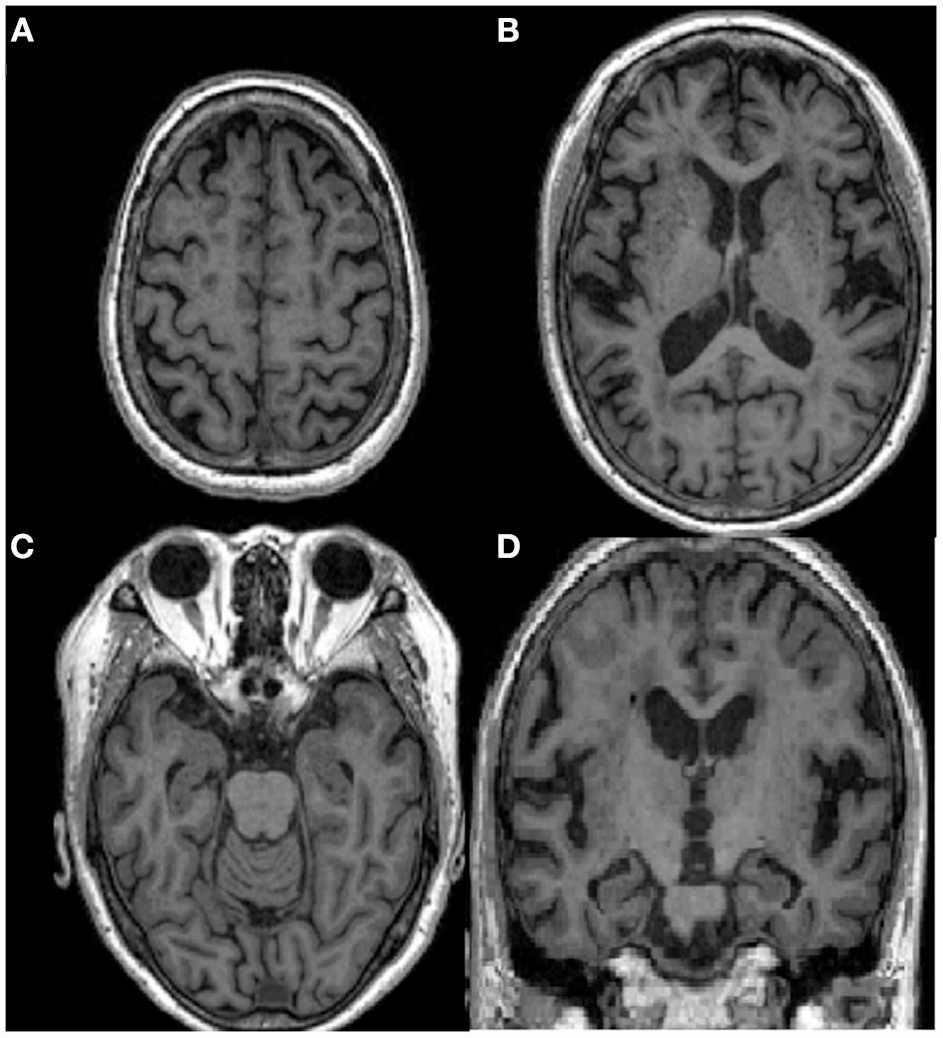
Brain MRI of the patient showing mild diffuse atrophy, compatible with her age. **(A–C)** Axial sections of T1 weighted images; **(D)** A coronal section.

**Figure 2:**
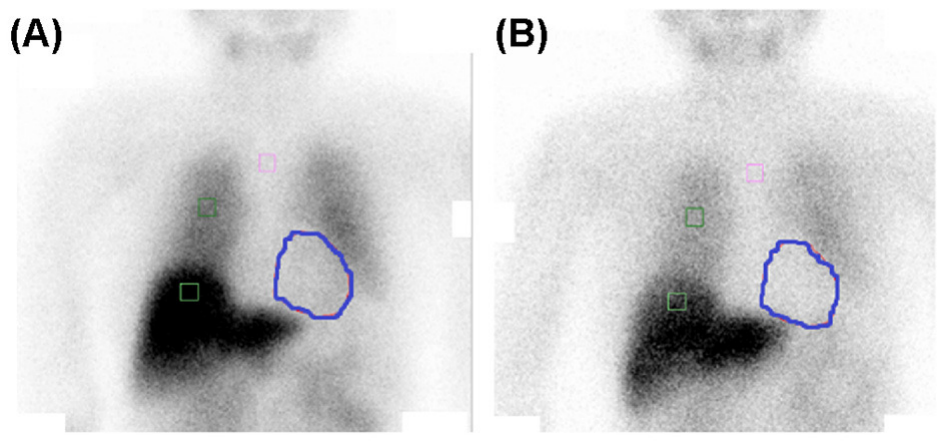
Myocardial accumulation of metaiodobenzylguanidine (123I-MIBG) is low [H/M = early: 1.72 **(A)**, delayed: 1.34 **(B)**]. The circled areas indicate heart.

On her first visit to our hospital, we did not observe any apparent depressive or manic symptoms. Neurological examinations revealed mild bradykinesia, mild rigidity of the left upper and lower limbs, and chronic constipation. The results of her cognitive assessment were as follows: Mini-Mental State Examination score was 20/30, a Japanese version of the Alzheimer's Disease Assessment Scale-cognitive subscale score was 10/70, the index of subtests of the digit span of Wechsler Adult Intelligence Scale-III was 5, and Mayo Fluctuation Questionnaire score was 5 out of 8, which indicated mild recent memory impairment, attention deficit, and cognitive fluctuation (Table 1). We conducted the Neuropsychiatric Inventory 12 to assess her neuropsychiatric symptoms. She scored 20 points, involving the categories of delusion (12/12) and apathy (8/12) (Table 1). Blood test results, including vitamins, thyroid function, and infections, were all normal. Brain magnetic resonance imaging revealed mild diffuse cortical atrophy and mild bilateral hippocampal atrophy, compatible with her age (Figure 1). Perfusion single photon emission computed tomography revealed mild hypoperfusion in the bilateral parietal lobe. Myocardial accumulation of metaiodobenzylguanidine (123I-MIBG) was low (H/M = early: 1.72, delayed: 1.34) (Figure 2). The aforementioned results indicated a probable diagnosis of DLB (12).

**Table 1:** The results of neuropsychological tests and the Neuropsychiatric Inventory (NPI).

	**Score**
Mini-Mental State Examination	20/30
Japanese version of the Alzheimer's Disease Assessment Scale-cognitive subscale score	10/70
Digit span of Wechsler Adult Intelligence Scale-III (index)	5
Mayo Fluctuation Questionnaire	5/8
Neuropsychiatric Inventory 12	
Delusions	12/12
Hallucinations	0
Agitation/aggression	0
Depression	0
Anxiety	0
Euphoria	0
Apathy	8/12
Disinhibition	0
Irritability/lability	0
Aberrant motor behavior	0
Sleep disturbances	0
Eating abnormalities	0

Following the examinations, she was prescribed 3 mg of donepezil, the dose of which was gradually increased to 10 mg. She was simultaneously prescribed 25 mg of quetiapine (at night) for the treatment of delusions. However, we soon discontinued quetiapine because of its adverse reactions, such as drowsiness and dizziness. We also prescribed brexpiprazole (1 mg/day) and risperidone (0.5 mg/day). However, their side effects, such as drowsiness, were extremely severe, without any amelioration of her delusion. While she did not refuse the medications, she still had no insight to her delusion. Moreover, she occasionally claimed to visit her family doctor following her discharge. We then prescribed blonanserin (4 mg/day) and continued it, with extremely mild side effects. After 2 weeks, her attitude to the delusional beliefs began to change. She gradually lost passion for her family doctor. Based on our suggestions, she changed her family doctor and was discharged from our hospital. During follow-up, she rarely talked about the previous doctor, who had been the subject of her delusion. She still lives alone, and her delusion has not recurred. She is currently on donepezil (10 mg/day) and a small amount of an antipsychotic agent, with coordination of the circumstances (non-pharmacotherapy).

## Discussion

De Clerambautlt's syndrome is based on the concept of erotomania proposed by de Clerambault in 1942 (13). He considered erotomania to manifest in the following two forms: (i) pure type and (ii) secondary type. Ellie et al. proposed the diagnostic criteria of the secondary form of de Clerambault's syndrome in 1985 as follows (14): (a) a delusional conviction of being in amorous communication with another person, (b) the other person being of a relatively higher rank, (c) the other person was the first to fall in love, (d) the other person was the first to make advances, (e) sudden onset, (f) the object of the amorous delusion remains unchanged, (g) the patient provides an explanation for the paradoxical behavior of the loved one, (h) chronic course, and (i) no hallucinations. The aforementioned case history and symptoms fulfilled all criteria.

Clinical features of the patient, such as gradually progressive cognitive deficit, fluctuating cognition, rigidity, hypersensitivity to antipsychotics, and low uptake in 123I-MIBG myocardial scintigraphy, indicated probable DLB (12). She experienced mild cognitive impairment, as revealed by several neuropsychological tests. Moreover, her activities of daily living were relatively preserved. Therefore, de Clerambault's syndrome supposedly appeared during the early stages of DLB. Psychiatric-onset DLB has increasingly gained attention in recent years. Some studies have reported sufficiently severe delusion requiring hospitalization during the early stages of DLB (15).

Sudden onset is one of the features of de Clerambault's syndrome. However, the above-mentioned case was unique in that the object of the delusion was also that of persecutory delusion, immediately before the manifestation of de Clerambault's syndrome. De Clerambault reported the possible association between the emotional state, including hypomania and the occurrence of the pure type of the syndrome. In addition, cases of the syndrome secondary to affective disorders were described more frequently in the manic state than in the depressive state (16). Our examinations failed to detect any emotional problems in the patient, including the results of NPI (Table 1). However, an emotional change, such as a very mild manic episode, might have existed around the emergence of the delusion. In contrast, the patients with de Clerambault's syndrome have been often reported to interpret usual situations, behaviors, or attitudes as proof of delusional love during the initial stages of the syndrome (17). The attitude of her family doctor toward her persecutory delusion might have been interpreted in the delusional context and become the cue for the appearance of the erotomanic delusion.

Schizophrenia is the most frequent disease that induces the secondary form of de Clerambault's syndrome. Nonetheless, other psychiatric disorders or conditions can also be the underlying causes. Of those diseases, only a few case reports have described de Clerambault's syndrome in patients with dementia (5). In each such report, cognitive impairment was relatively mild. Moreover, some studies have reported the association between a deficit of frontal lobe function and the appearance of delusions (5). The profile of cognitive impairment in the aforementioned case was compatible with the features suggested in previous reports. According to Kraepelin, patients suffering from erotomania usually have preserved intellectual function (17). Similarly, the occurrence of de Clerambault's syndrome may require relatively preserved cognitive function in patients with dementia. In addition, the neural correlates of the other delusions in dementia have been investigated in previous studies, some of which established the association between delusions and hypoperfusion or a functional deficit in the frontal lobe (18, 19). The same could be true of de Clerambault's syndrome in patients with dementia. This necessitates further investigations of the neural correlates of de Clerambault's syndrome.

In patients with dementia, the use of antihypertensives has been reported to be a risk of delusions. Since the present patient lived alone for a long time, her detailed medication history was uncertain. Although she seemed to have been prescribed medications for hypertension and constipation nearly 20 years according to information from herself and her daughter, we could not rule out the possibility that some medication influenced the appearance of the erotomanic delusion.

Approximately 60% of patients with DLB have delusions (5). More than 50% of these patients develop delusions in the mild stages (clinical dementia rating, 0.5) (20). There have been some reports on patients with DLB and unusual delusions, such as Othello syndrome (11), delusional parasitosis (21, 22) and delusion of duplication (23). In particular, Othello syndrome, characterized by delusional beliefs of infidelity of a partner, was known to be found in as much as 26.3% of DLB (11). Although De Clerambault's syndrome and Othello syndrome have a common “sexual” theme, few reports of De Clerambault's syndrome have been reported in DLB. Inappropriate sexual behavior is relatively common in people with dementia (24, 25), and the dysfunction of frontal lobes, cortico-striatal circuit and dopaminergic pathway are known to be the bases of inappropriate sexual behavior (26, 27). However, de Clerambault's syndrome has been more associated to manic state than to depressive state (16) and depressive state is known to be more common in patients with neurodegenerative dementias including DLB (20). On the other hand, Othello Syndrome has been reported to be more associated with depression than bipolar disorder (25). The relevance to mood disturbances may account for the rare occurrence of de Clerambault's syndrome in patients with neurodegenerative dementias including DLB. Further studies are needed to prospectively investigate the association between senile onset delusions, particularly unusual delusions and DLB diagnosis. This can be attributed to previous reports on delusions being the initial symptoms in the prodromal stage of DLB (15) and their development during the mild stages of DLB (20, 28).

The treatment of de Clerambault's syndrome is relatively difficult (17). Most reports on its pharmacological treatment have suggested the usefulness of antipsychotic agents, including pimozide (12, 18), risperidone (29), and olanzapine (30). Quetiapine (50 mg/day) failed to remit the delusion in a patient with AD (6). In this case, quetiapine, risperidone, and brexpiprazole failed to exert their effectiveness because of adverse reactions. However, the impact of these agents on de Clerambault's syndrome in such patients is unclear, because the duration of administration was not long enough to judge their actual usefulness. In this case, blonanserin did not produce severe side effects and was effective against delusions in low dosage. Considering the hypersensitivity to antipsychotic drugs in patients with DLB (13), a small dosage might be recommended initially and, if necessary, the dose can be gradually increased, with a careful observation of the side effects. Further studies are required to investigate the pharmacological treatment of patients with DLB and de Clerambault's syndrome.

The aforementioned case report revealed that de Clerambault's syndrome could appear during the early stages of disease in patients with DLB. Previous studies have reported the occurrence of relatively rare delusions during the early stages of DLB. This necessitates further accumulation of knowledge about delusions in patients with DLB for an early diagnosis.

## Data Availability Statement

The raw data supporting the conclusions of this article will be made available by the authors, without undue reservation.

## Ethics Statement

Written informed consent was obtained from the patient and her family for the publication of any potentially identifiable images or data included in this study.

## Author Contributions

TS conducted treated the patient during admission, collected the data, and wrote the initial draft of this article. MI and YS conducted the outpatient treatment. MI, MH, and YS offered advice for the treatment and participated in the discussion of the results. All authors contributed to the article and approved the submitted version.

## Conflict of Interest

The authors declare that the research was conducted in the absence of any commercial or financial relationships that could be construed as a potential conflict of interest.
